# Establishment of Novel Genotoxicity Assay System Using Murine Normal Epithelial Tissue-Derived Organoids

**DOI:** 10.3389/fgene.2021.768781

**Published:** 2021-11-18

**Authors:** Masami Komiya, Rikako Ishigamori, Mie Naruse, Masako Ochiai, Noriyuki Miyoshi, Toshio Imai, Yukari Totsuka

**Affiliations:** ^1^ Department of Cancer Model Development, National Cancer Center Research Institute, Tokyo, Japan; ^2^ Department of Animal Experimentation, National Cancer Center Research Institute, Tokyo, Japan; ^3^ Food Environment Research Center, Graduate Division of Nutritional and Environmental Sciences, University of Shizuoka, Shizuoka, Japan; ^4^ Graduate School of Integrated Pharmaceutical and Nutritional Sciences, University of Shizuoka, Shizuoka, Japan; ^5^ Laboratory of Environmental Toxicology and Carcinogenesis, School of Pharmacy, Nihon University, Chiba, Japan

**Keywords:** murine organoids, *gpt* delta mice, PhIP, acrylamide, base substitution

## Abstract

Short-/middle-term and simple prediction studies for carcinogenesis are needed for the safety assessment of chemical substances. To establish a novel genotoxicity assay with an *in vivo* mimicking system, we prepared murine colonic/pulmonary organoids from *gpt* delta mice according to the general procedure using collagenase/dispase and cultured them in a 3D environment. When the organoids were exposed to foodborne carcinogens—2-amino-1-methyl-6-phenylimidazo(4,5-*b*)pyridine (PhIP) and acrylamide (AA)—in the presence of metabolic activation systems, mutation frequencies (MFs) occurring in the *gpt* gene dose-dependently increased. Moreover, the mutation spectrum analysis indicated predominant G:C to T:A transversion with PhIP, and A:T to C:G and A:T to T:A transversion with AA. These data correspond to those of a previous study describing *in vivo* mutagenicity in *gpt* delta mice. However, organoids derived from the liver, a non-target tissue of PhIP-carcinogenesis, also demonstrated genotoxicity with a potency comparable to colonic organoids. Organoids and PhIP were directly incubated in the presence of metabolic activation systems; therefore, there was a lack of organ specificity, as observed *in vivo*. Additionally, PhIP-DNA adduct levels were comparable in hepatic and colonic organoids after PhIP exposure. Taken together, the organoids prepared in the present study may be helpful to predict chemical carcinogenesis.

## Introduction

Safety evaluation is required to develop chemical substances, such as food additives and pharmaceuticals (Yamada and Honma, 2018). As many carcinogens are genotoxic, genotoxicity tests are conducted to evaluate the safety of chemical substances. The Ames test, micronucleus test, chromosome aberration test, and gene mutation tests targeting a reporter gene have been widely used as simple *in vitro* genotoxicity tests (Gatehouse, 2012; Steinberg, 2017; Mortelmans, 2019; White et al., 2019). However, these systems have various limitations, including the lack of xenobiotic metabolism required for activation and detoxification. Moreover, a single culture of tissue-resident cells is used in *in vitro* assays to evaluate the genotoxicity of chemicals; however, cell-cell interactions between tissue-resident cells or immune cells are critical. Therefore, it is difficult to extrapolate the safety of chemical substances to humans using only these ordinal *in vitro* tests. Since *in vivo* safety evaluation tests such as carcinogenesis tests require long-term observation using large numbers of experimental animals, it is considered necessary to develop an *in vitro* method that can predict carcinogenicity in the short to medium term, taking into account the 3Rs (replacement, reduction and refinement). It is desirable to develop *in vitro* genotoxicity assessment systems that mimic tissues and can be extrapolated to humans.

To meet these demands, a variety of novel toxicity assessment methods have been developed. We have established an *in vivo* simulation co-culture assay platform for testing the genotoxicity of nanosized materials (Kato et al., 2017; Fukai et al., 2018). We induced DNA damage in A549 cells, which are derived from human lung cancer, by exposure to a nanomaterial, kaolin. In these cells, co-culture with kaolin-exposed RAW264 cells, derived from murine macrophage-like immune cells, markedly increased the magnitude of DNA damage formation (Kato et al., 2017). Moreover, we established a mechanism-based assay to evaluate the genotoxicity of multi-walled carbon nanotubes (MWCNTs) under conditions simulating an *in vivo* situation, featuring a co-culture system of murine lung resident cells (GDL1) and immune cells (RAW264.7). Mutation frequencies induced in GDL1 by the MWCNTs were significantly greater with the co-existence of RAW264.7 than in its absence. The mutation spectra observed in GDL1 co-cultured with RAW264.7 cells were different from those observed in GDL1 cultured alone but similar to those observed in the lungs of mice exposed to the MWCNTs *in vivo* (Fukai et al., 2018). Therefore, co-culture of tissue-resident cells and immune cells may be a suitable *in vitro* assay to evaluate genotoxicity, as it adequately simulates *in vivo* conditions.

Similar to the co-culture model, organoids are also considered systems that mimic living organisms. Many studies have used organoids to study carcinogenesis pathways at the molecular level (Onuma et al., 2013; Matsuura et al., 2020; Idris et al., 2021). We recently established an *ex vivo* model for chemical carcinogenesis using normal mouse tissue-derived organoids and demonstrated that this model could be applied to detect early molecular events (Naruse et al., 2020).

**FIGURE 1:**
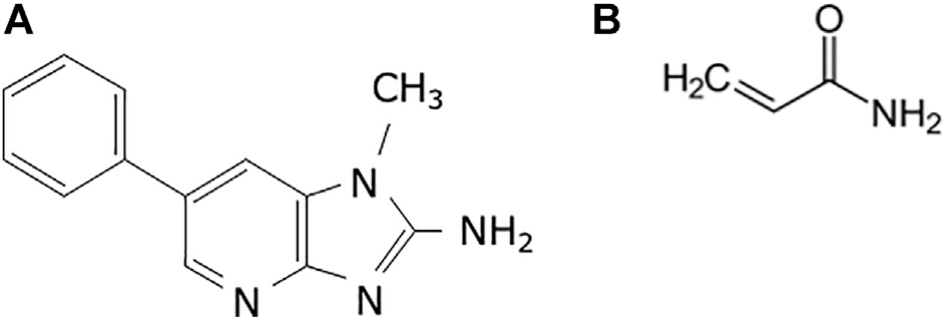
Chemical structures used in the study. **(A)** 2-Amino-1-methyl-6-phenylimidazo(4,5-*b*)pyridine (PhIP) and **(B)** acrylamide (AA).

The present study aimed to investigate the validity of using organoids generated from experimental animals to evaluate mutagenicity. We selected organoids derived from guanine phosphoribosyl transferase (*gpt*) delta mice (background strain C57BL/6J) (Nohmi et al., 2000). In this mouse model, point mutations and deletions are analyzed by *gpt* and Spi-selections, respectively, and are widely used in the field of environmental mutagenicity, including 2-amino-1-methyl-6-phenylimidazo [4,5-*b*]pyridine (PhIP) and acrylamide (AA) (Figure 1) (Masumura et al., 1999; Masumura et al., 2000; Ishii et al., 2015). Both chemicals are known as foodborne carcinogens and are responsible for the development of some human cancers (de Conti et al., 2019; Adani et al., 2020; Bellamri et al., 2021; Le Marchand, 2021). Moreover, PhIP is produced by Maillard reactions between glucose, phenylalanine, and creatinine and is the most abundant heterocyclic amine produced during the cooking of meat and fish (Felton et al., 2000). In F344 rats, PhIP can induce tumors in the colon and prostate in males, and in the mammary glands of female rats. It also induced colon tumors in the dextran sulphate sodium-induced colitis mouse model (Tanaka et al., 2005; Bellamri et al., 2021). In contrast, AA is produced by the heating of carbohydrate-rich foods such as potatoes at high temperatures. AA is a known carcinogen that targets the mouse lung (Beland et al., 2013). As both chemicals are produced during cooking and contaminate ordinary foods, humans are continuously exposed to these carcinogenic compounds. Therefore, in the present study, organoids derived from the normal colon and lungs were established from *gpt* delta mice, and mutagenicity was evaluated after multiple PhIP and AA exposures. Additionally, we investigated mutagenicity in the liver, a non-target tissue for PhIP-carcinogenesis.

## Materials and Methods

### Chemicals

PhIP-HCl was purchased from the Nard Institute (Osaka, Japan), and its purity was confirmed to be >99% by HPLC. AA was obtained from Sigma Aldrich (St. Louis, MO, United States). Nuclease P1 was purchased from Wako Pure Chemical Industries (Osaka, Japan). Phosphodiesterase I was purchased from Worthington. Nicotinamide, bovine spleen phosphodiesterase II, DNase I, and bacterial alkaline phosphatase type III (*E. coli*) were purchased from Sigma Aldrich (St. Louis, MO, United States). Difco™ Nutrient Broth and Cofactor-I were acquired from Becton, Dickinson and Company (Sparks, MD, United States), and Oriental Yeast Co., Ltd (Tokyo, Japan), respectively. The S9 mix was purchased from the IEDA Trading Corporation (Tokyo, Japan). All other chemicals used in the study were of analytical grade and were purchased from Wako Pure Chemical Industries.

### Animals

The *gpt* delta mice were purchased from Japan SLC (Shizuoka, Japan). The *gpt* delta mice harbor approximately 80 copies of *lambda* EG10 DNA on chromosome 17 on a C57BL/6J background (Nohmi et al., 2000). The mice used to establish the lung, and liver organoids were male. All animal experiments were performed following protocols approved by the Committee for the Ethics of Animal Experimentation of the National Cancer Center (approval protocol no. T17-029-M05).

### Organoid Culture

The lungs, liver, and colon were dissected from five-week-old *gpt* delta mice. Previously reported procedures for organoid culture of the colon and lung were used in the present study (Onuma et al., 2013; Maru et al., 2019; Naruse et al., 2020). For the generation of liver organoids, advanced DMEM/F12 (Thermo Fisher Scientific, Waltham, MA) supplemented with 10 ng/ml murine EGF (Wako Pure Chemical, Osaka, Japan), 10 μM Y-27632 (Wako), 0.5 μΜ CultureSure® A-83-01 (Wako), 3 μΜ CHIR-99021(Focus Biomolecules, Plymouth Meeting, PA), 5 mM HEPES (Thermo Fisher Scientific), ITS-G supplement (Wako), 0.05% albumin D-PBS(-) solution (Wako), 10 mM CultureSure® Nicotinamide (Wako), 30 μg/mL l-proline (Sigma Aldrich, St. Louis, MO), 39.2 ng/ml dexamethasone (Sigma Aldrich), and 1 mM L-ascorbic acid 2-phosphate trisodium salt (Wako) were used. Cells were further dissociated by pipetting into single cells, seeded on 65 μl of Matrigel (Corning, Bedford, MA) per well in a 12-well plate, and incubated 16 h at 37°C. One day later, the supernatant containing dead cells and debris was removed, and viable cells attached to Matrigel were covered with an additional 75 μl of Matrigel and overlaid with media to resume 3D culture (Matrigel Bilayer Organic Culture; MBOC) (Alic et al., 2011). Passaging was conducted every 5–10 days at a dilution of 1:3. In each passage, organoids were directly collected with a cell scraper, washed with PBS, and dissociated into single cells using Accumax (Innovative Cell Technologies, San Diego, CA) for 12 min at room temperature and vigorous pipetting. Dissociated cells were seeded on Matrigel for overnight incubation, and only attached cells were subjected to subsequent 3D culture as described previously (Onuma et al., 2013; Maru et al., 2019; Naruse et al., 2020).

### Chemical Treatments

Treatment with PhIP at 0, 5, and 10 μM for the colon- and liver-derived organoids and AA at 0, 0.28, and 1.4 mM for the lung-derived organoids for 24 h was repeated three times after passaging the organoids. The same concentrations used in our previous report (Naruse et al., 2020) were chosen for AA in the present study. On the other hand, cell viabilities after a single exposure of PhIP at the concentrations of 2, 5, and 10 μM were 150 ± 46, 113 ± 60, and 73 ± 17%, respectively (data obtained from three independent studies). Thus, the concentrations of PhIP were selected at 5 and 10 μM in the present study. Chemical treatment at each concentration was performed by mixing with S9 mix (50 μg/ml of S9 protein), and the cell number of organoids corresponded to 1.0 × 10^5^ cells/well in a 12-well plate.

### *gpt* Mutation Assay

High molecular weight genomic DNA from the colonic, hepatic, and pulmonary organoids was extracted using RecoverEase DNA Isolation Kit (Agilent Technology, United States), according to the manufacturer’s instructions. *Lambda* EG10 phages were rescued using Transpack Packaging Extract (Stratagene, La Jolla, CA, United States). The *gpt* mutation assay was performed according to previously described methods (Fukai et al., 2018). Briefly, *E. coli* YG6020 was infected with the phage and spread on M9 salt plates containing chloramphenicol (Cm) and 6-thioguanine (6-TG) and incubated for 72 h at 37°C to select colonies with a plasmid carrying the gene encoding chloramphenicol acetyltransferase, as well as mutated *gpt*. The 6-TG–resistant isolates were cultured overnight at 37°C in LB broth containing 25 mg/ml of Cm, harvested by centrifugation (7,000 rpm, 10 min), and stored at −80°C. Mutational spectra of 6-TG coding sequences were determined using PCR and direct sequencing, and a 739-bp DNA fragment containing *gpt* was amplified by PCR as described previously (Fukai et al., 2018). Sequence analysis was performed using Eurofins Genomics software (Tokyo, Japan).

### PhIP-DNA Adduct Analysis

The murine colonic / hepatic organoids were exposed to PhIP at a concentration of 5 μM in the presence of S9 mix for 24 h using the procedure described above. Control samples were prepared by treatment with vehicle (0.15% DMSO) instead of PhIP. DNA was extracted from the colonic-/hepatic organoids (approximately 4 × 10^5^ cells) using Gentra® Puregene™ tissue kit (QIAGEN, Valencia, CA) and stored at −80°C until further use. We prepared three independent samples for each group. The amount of extracted DNA was individually insufficient for adduct analysis. Still, we pooled all three samples and digested 15–35 μg of DNA with DNase I, nuclease P1, and alkaline phosphatase, as described previously (Ishino et al., 2015). The amount of *N*-(deoxyguanosine-8-yl)-2-amino-1-methyl-6-phenylimidazo[4,5-*b*]pyridine (dG-C8-PhIP) was measured following the procedure described previously with a slight modification of LC-MS conditions (Iwasaki et al., 2014). Briefly, LC-MS analysis was performed using Shimadzu Prominence LC system interfaced with Triple TOF6600 mass spectrometer (SCIEX, Massachusetts, United States) in the product ion (PI) mode. The HPLC conditions were as follows: column = Synergi^TM^ Fusion-RP (2.5 mm particle size, 2.0 × 100 mm; Phenomenex, California, United States); flow rate = 0.4 ml/min; and solvent system = linear gradient from 2.5 to 85% acetonitrile in 10 mM ammonium acetate (pH 5.3) for 30 min. The dG-C8-PhIP was analyzed in PI mode with the major fragment ions *m/z* 490.1→374.1 corresponding to the loss of the deoxyribose moiety (PhIP-G) with a cone voltage of 35 V and collision energy of 20 eV. Quantification was performed using a standard curve of authentic dG-C8-PhIP.

### Real-Time PCR Analysis

Total RNA was isolated from the lung organoids and lungs of *gpt* delta mice using ISOGEN (Nippon Gene, Tokyo), and aliquots (100 ng) in a final volume of 20 μL were used for the synthesis of cDNA using SuperScript IV VILO Master Mix (Thermo Fisher Scientific) and oligo primers. Real-time PCR was performed using CFX96^TM^ (BIO-RAD) with KAPA SYBR FAST qPCR Master Mix (2x) (KAPA BIOSYSTEMS) according to the manufacturer’s instructions. Primers for the mouse *Cyp2e1* (5′-CAG​GAG​TAC​AAG​AAC​AAG​GGG and 3′- CAG​AAA​GGT​AGG​GTC​AAA​AGG) and *Gapdh* (5′-TCG​CTC​CTG​GAA​GAT​GGT​GA and 3′-TGA​GAA​CGG​ATT​TGG​CCG​TA) were used.

The PCR conditions were as follows: 95°C for 10 min, followed by 42 cycles of 95°C for 15 s, 60°C for 10 s, and 72°C for 20 s. The PCR ended with a final elongation step at 72°C for 5 s. To assess the specificity of each primer set, the amplicons generated from PCR were analyzed for melting curves.

### Statistical Analysis

For statistical comparison of the data of the experimental and control groups from the *gpt* mutation and *Cyp2e1* expression analysis, expressed as means ± standard deviations, we first performed the *F* test. If the *F* test revealed that the variances were unequal, the *p* was determined using Welch’s *t*-test. If not, the Student’s *t*-test was used instead. In mutation spectrum analysis, *p* was determined using Fisher’s exact test according to Carr and Gorelick (Carr and Gorelick, 1996). In any case, *p* < 0.05 indicated statistical significance.

## Results

### Decrease in Organoids Cell Number and Change in Morphology due to PhIP or AA Exposure

**FIGURE 2:**
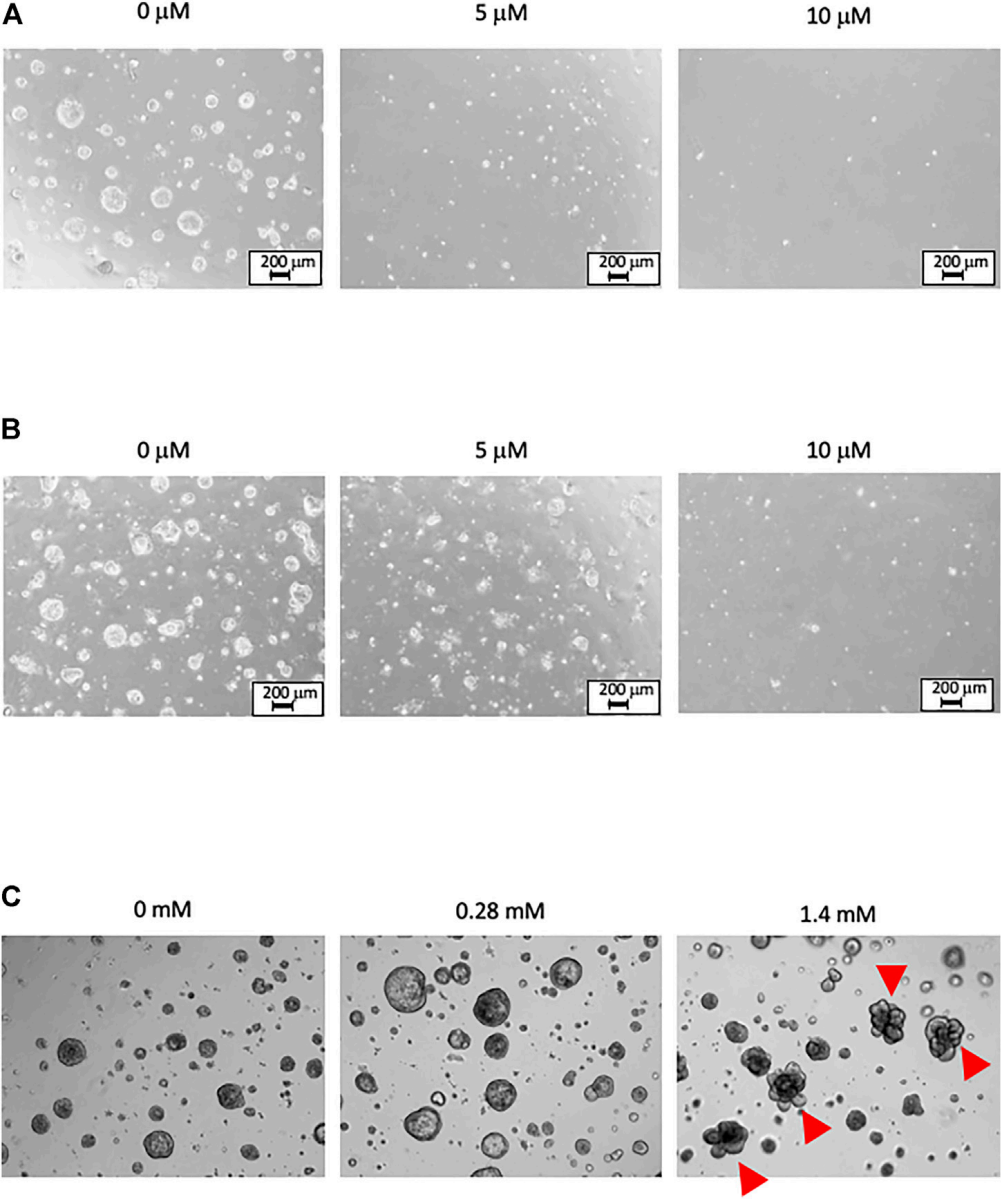
Phase-contrast images of organoids under Matrigel-based 3D culture. The images are obtained 1 day after the third treatment. **(A)** The PhIP-treated colonic organoids. No obvious change is observed in the vehicle treatment (0 μM). Exposure to PhIP increased the number of dead cells in a concentration-dependent manner. **(B)** The PhIP-treated hepatic organoids show a trend similar to that observed for colonic organoids. **(C)** The AA-treated lung organoids show no significant changes at the low-concentration of AA treatment. Morphological changes are observed in several lung-derived organoids at the high-concentration of AA (indicated by arrowheads). All images of **(C)** are shown at the original magnification x 20.

Images of organoids in Matrigel-based 3D culture are shown in Figure 2. The organoids were repeatedly treated with PhIP and AA. No evident change was observed upon vehicle treatment; however, the number of dead cells was increased in the PhIP-treated colonic and hepatic organoids in a concentration-dependent manner (Figures 2A,B). In contrast, no significant changes were observed with the initial AA treatment; whereas, several morphological changes were observed in the lung-derived organoids at high concentrations of AA (Figure 2C).

### *gpt* Mutations in the Murine Colonic- and Hepatic Organoids Exposed to PhIP

To determine the mutagenic effects of PhIP in the colon, which is a target organ for PhIP-carcinogenesis, the colonic-organoid was treated with PhIP at 5 and 10 μM concentrations, repeated three times. The data are summarized in Table 1. Mutation frequencies (MFs) observed in the colonic organoids were significantly increased by both low and high concentrations of PhIP (11.8- and 12.9-fold, respectively) than those of the vehicle control.

**TABLE 1:** Summary of MFs in the colonic organoids of *gpt* delta mice.

Treatment	Organoid ID	Number of colonies		MF (× 10^−6^)	Average MF (×10^−6^)*
		Mutant	Total		
Control	1	1	402,000	2.5	3.8 ± 4.3
	2	1	355,500	2.8	
	3	0	39,000	0.0	
	4	1	100,500	10.0	
PhIP 5 μM	1	5	187,500	26.7	45.0 ± 17.3
	2	16	337,500	47.4	
	3	28	459,000	61.0	
PhIP 10 μM	1	26	453,000	57.4	49.2 ± 14.8
	2	20	345,000	58.0	
	3	8	249,000	32.1	

*Mean ± SD.

The classes of *gpt* mutations observed in the colonic organoids are summarized in Table 2. As only a limited number of spontaneous mutant clones (*n* = 3) were observed in the present study, previously published data are included in Table 2 (Masumura et al., 2000). Point mutations were predominant. Among the mutation profiles observed in the *gpt* coding sequence in the colonic organoids, the proportion of G:C to T:A transversion was significantly higher in PhIP-exposed clones than that in control clones (Table 2). Additionally, the proportions of G:C to A:T transition and G:C to C:G transversion and deletions were also increased in PhIP-exposed clones than in control clones, similar to those observed in an *in vivo* study (Table 2; Masumura et al., 2000).

**TABLE 2:** Classification of *gpt* mutations isolated from the colonic organoids with/without PhIP-treatment.

	Type of mutation		Control		Control-2^a^		PhIP		PhIP-2^a^	
			Number of mutants (%)	Specific MF^c^ (10^–6^)	Number of mutants (%)	Specific MF^c^ (10^–6^)	Number of mutants (%)	Specific MF^c^ (10^–6^)	Number of mutants (%)	Specific MF^c^ (10^–6^)	*p* value^b^
Base substitution	Transition	G:C to A:T	0 (0)	0	31 (43.1)	2.62	13 (37.1)	6.40	14 (14.1)	15.70	0.019^d^, 0.005^e^
		A:T to G:C	0 (0)	0	8 (11.1)	0.67	0 (0)	0	0 (0)	0	−^d^, 0.241^e^
	Transversion	G:C to T:A	3 (100)	3.34	19 (26.4)	1.60	15 (42.9)	7.39	52 (52.5)	58.30	0.226^d^, 0.0000012^e^
		G:C to C:G	0 (0)	0	0 (0)	0	1 (2.9)	0.49	13 (13.1)	14.58	0.516^d^, 0.016^e^
			A:T to T:A	0 (0)	0	4 (5.6)	0.34	0 (0)	0	1 (1.0)	1.12	−^d^, 0.407^e^
		A:T to C:G	0 (0)	0	3 (4.2)	0.25	0 (0)	0	0 (0)	0	−^d^, 0.473^e^
	Insertion		0 (0)	0	4 (5.6)	0.34	0 (0)	0	1 (1.0)	1.12	−^d^, 0.407^e^
	Deletion		0 (0)	0	3 (4.2)	0.25	6 (17.1)	2.95	15 (15.1)	16.81	0.1114^d^, 0.00001^e^
	Others		0 (0)	0	0 (0)	0	0 (0)	0	3 (3.0)	3.36	−^d^, −^e^
	Total		3 (100)	3.34	72 (100)	6.08	35 (100)	17.2	99 (100)	111.0	1.413 × 10^−9^ ^d^, 1.932 × 10^−61^ ^e^

a Data are from Masumura et al.

b *p* values were determined using Fisher’s exact test according to Carr and Gorelick.

c Specific MFs, were calculated by multiplying the total mutation frequency by the ratio of each type of mutation to the total mutation.

d PhIP vs. control.

e PhIP vs. control-2.

**FIGURE 3:**
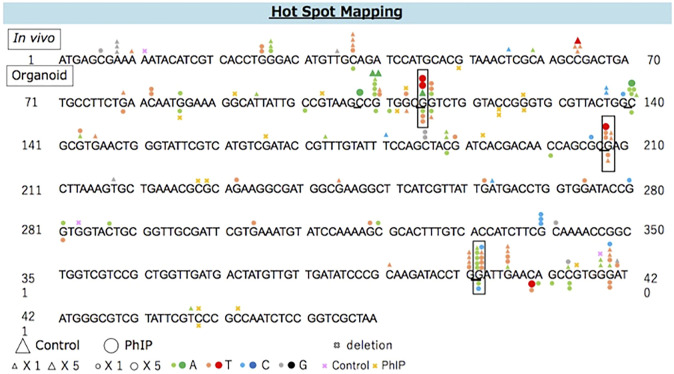
The distribution of spontaneous and PhIP-induced mutations in the coding region of *gpt* in the colonic-organoids. Mutations obtained from *in vivo* data (Masumura et al., 2000) are shown above, and those obtained from the present study are shown below the wild-type sequence. Triangle: spontaneous point mutation (small, single mutation; large, five mutations). Circle: PhIP-induced point mutation (small, single mutation; large, five mutations). Colors indicate that nucleobase changed to adenine (green), thymine (red), cytosine (blue), guanine (black). Pink and yellow marks indicate spontaneous and PhIP-induced deletion mutations, respectively.

The distribution of PhIP-induced and spontaneous mutations in the coding region of *gpt* is shown in Figure 3. To compare the organoid and *in vivo* studies, previous data are also depicted in Figure 3. Base substitutions were spread throughout the coding region, with a preference for some sites. Six out of 35 PhIP-induced mutations with C to T transitions occurred at position 409, which was the hot spot for the action of PhIP in the present study. Moreover, single base substitutions were also observed at positions 115, 401, 413, and 418 at high frequencies. Of these, two hot spots (positions 115 and 401) corresponded to the PhIP hot spots observed in the *in vivo* study (Masumura et al., 2000). The G to T transversions were dominant (40%), followed by G to A transitions (34%).

Further, we analyzed the mutation frequency in the liver-derived organoids exposed to PhIP. The results of the *gpt* mutation assay are summarized in Table 3. Although, liver is a non-target tissue for PhIP-carcinogenesis, PhIP significantly induced MFs in hepatic organoids, compared to colonic organoids (Table 3). However, the background MF of the hepatic organoids was much higher than that in the colonic organoids (61.7 × 10^–6^ vs. 3.8 × 10^–6^).

**TABLE 3:** Summary of MFs in the hepatic organoids of *gpt* delta mice.

Treatment	Number of colonies		MF (×10^−6^)
	Mutant	Total	
Control	5	81,000	61.7
PhIP 5 μM	79	183,000	431.7
PhIP 10 μM	163	283,500	575.0

Point mutations predominated in the hepatic organoids, but the proportion of G:C to C:G transversion was high (36%), followed by proportion of G:C to T:A (25%) transversion, which was slightly different from that of the colonic organoids. A majority of the G:C to C:G transversion occurred equally at positions 115 and 143, and the former position 115) corresponded to one of the PhIP-mutational hot spots (Supplementary Figure S1). Additionally, position 208 with G to T transversion corresponded to one of the PhIP-mutational hot spots previously reported in an *in vivo* study (Masumura et al., 2000).

### Analysis of PhIP-DNA Adducts in the Murine Colonic- and Hepatic Organoids

**FIGURE 4:**
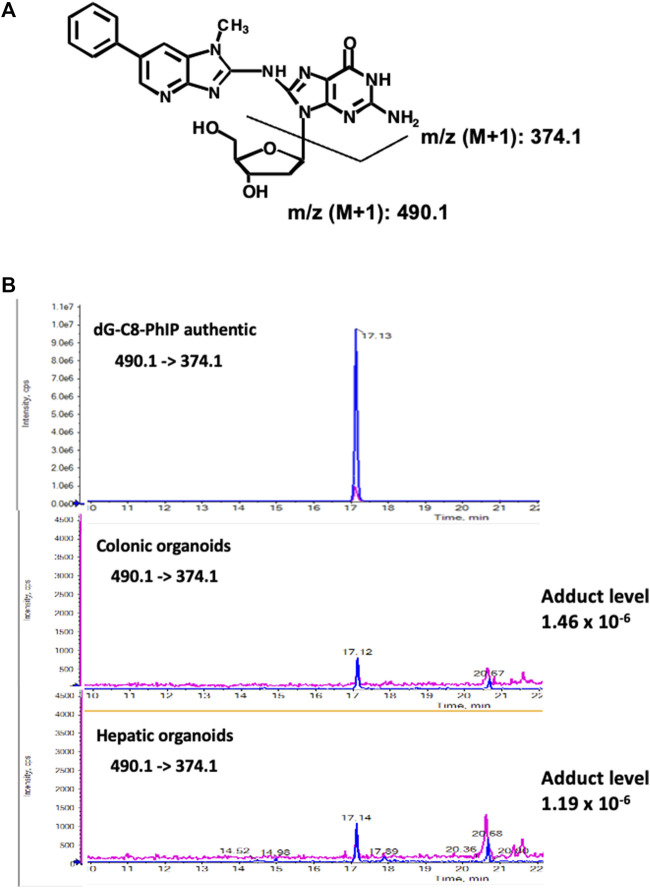
Chemical structures of dG-C8-PhIP and typical LC–ESI-MS/MS chromatograms of authentic dG-C8-PhIP and DNA samples obtained from the murine colonic- and hepatic organoids after 24 h exposure to PhIP at 5 μM concentration in the presence of S9 mix, measured in the product ion mode with m/z 490.1 → 374.1 (PhIP-Guanine). **(A)** Chemical structures of dG-C8-PhIP and deduced fragmentations. **(B)** LC–ESI-MS/MS chromatograms of authentic dG-C8-PhIP and DNA samples obtained from the murine colonic- and hepatic organoids. The peaks that correspond to dG-C8-PhIP are indicated by arrows.

As a similar induction rate of MFs was observed in the colonic and hepatic organoids after exposure to PhIP, we analyzed the formation of PhIP-DNA adduct, a promutagenic substance. To obtain sufficient DNA samples for adduct analysis, we combined three–five batches to prepare pooled colonic- and hepatic-organoid samples. A peak with m/z 490.1 (PhIP-dG) → 374.1 (PhIP-G) that corresponded to PhIP-DNA adduct was detected at the retention time same as authentic dG-C8-PhIP in both the colonic and hepatic organoids, and their adduct levels were almost comparable, i.e., 1.46 (colonic) and 1.19 (hepatic) adducts per 10^–6^ nucleotides (Figure 4).

### *gpt* Mutations in the Murine Pulmonary Organoids Exposed to AA

To confirm the mutagenic effects of AA in the lungs, a target organ for AA-carcinogenesis, the pulmonary organoid was treated three times with AA at 0.28 and 1.4 mM concentration. The results are summarized in Table 4. The MFs observed in the low-concentration AA group were similar to that in the vehicle control group. Nevertheless, a significant increase (approximately four-times) was observed in the high-concentration group than in the vehicle control group. The classes of *gpt* mutations observed in the pulmonary organoids, along with the previously published data from an *in vivo* study (Ishii et al., 2015), are summarized in Table 5. Among the mutation profiles that were observed for the 6-TG coding sequence in the lung-derived organoids, the proportion of G:C to A:T transition, A:T to T:A, and A:T to C:G transversions and deletions were significantly elevated in the AA-exposed clones than that in vehicle control clones (Table 4). Of these, the proportions of A:T to C:G and A:T to T:A transversions were also increased in the *gpt* delta mice after administration of AA (Ishii et al., 2015).

**TABLE 4:** Summary of MFs in the pulmonary organoids of *gpt* delta mice.

Treatment	Organoid ID	No. of colonies		MF (×10^6^)	Average MF (×10^−6^)*
		Mutant	Total		
Control	1	2	265,500	7.533	7.42 ± 2.70
	2	1	214,500	4.662	
	3	3	298,500	1.005	
AA 0.28 mM	1	0	300,000	0.000	7.19 ± 6.44
	2	2	219,000	9.132	
	3	3	241,500	12.42	
AA 1.4 mM	1	7	282,000	24.82	30.76 ± 20.84
	2	4	295,500	13.54	
	3	11	204,000	53.92	

*Mean ± SD.

**TABLE 5:** Classification of *gpt* mutations isolated from the pulmonary organoids with/without AA treatment.

	Type of mutation		Control		Control-2^a^		AA		AA-2^a^		*p* value^b^
			Number of mutants (%)	Specific MF^c^ (10^–6^)	Number of mutants (%)	Specific MF^c^ (10^–6^)	Number of mutants (%)	Specific MF^c^ (10^–6^)	Number of mutants (%)	Specific MF^c^ (10^–6^)	
Base substitution	Transition	G:C to A:T	1 (16.7)	1.28	6 (60.0)	0.15	8 (16.0)	5.19	8 (16.7)	2.28	0.154^d^, 0.021^e^
		A:T to G:C	0 (0)	0	0	0	0 (0)	0	6 (12.5)	1.71	−^d^, −^e^
	Transversion	G:C to T:A	2 (33.3)	2.57	1 (10.0)	0.02	4 (8.0)	2.59	6 (12.5)	1.71	0.418^d^, 0.177^e^
			0 (0)	0	1 (10.0)	0.03	0 (0)	0	2 (4.2)	0.57	−^d^, 0.521^e^
												G:C to C:G
		A:T to T:A	0 (0)	0	0	0	3 (6.0)	1.95	3 (6.3)	0.85	0.218^d^, 0.007^e^
		A:T to C:G	0 (0)	0	0	0	5 (10.0)	3.24	2 (4.2)	0.57	0.112^d^, 0.0005^e^
	Insertion		0 (0)	0	0	0	3 (6.0)	1.95	0 (0)	0	0.218^d^, 0.007^e^
	Deletion		3 (50.0)	3.85	2	0.06	27 (54.0)	17.51	21 (43.7)	5.98	0.006^d^, 3.736 × 10^−14^ ^e^
	Others		0 (0)	0	0	0	0 (0)	0	0 (0)	0	−^d^, −^e^
	Total		6 (100)	7.71	10 (100)	0.26	50 (100)	32.43	48 (100)	1.36	0.061^d^, 1.197 × 10^−11^ ^e^

a Data are from Ishi et al.

b *p* values were determined using Fisher’s exact test according to Carr and Gorelick.

c Specific MFs, were calculated by multiplying the total mutation frequency by the ratio of each type of mutation to the total mutation.

d AA vs. control.

e AA vs. control-2.

**FIGURE 5:**
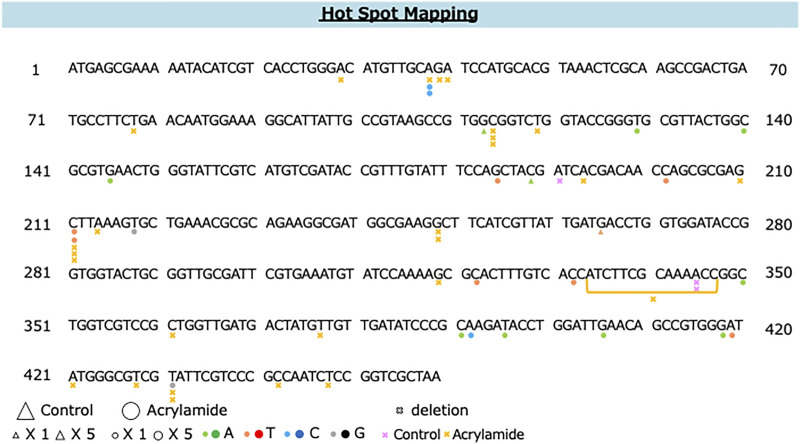
The distribution of spontaneous and AA-induced mutations in the coding region of *gpt*. Mutations obtained from the present study are shown below the wild-type sequence. Triangle: spontaneous point mutation (small, single mutation; large, five mutations). Circle: AA-induced point mutation (small, single mutation; large, five mutations). Colors indicate that nucleobase changed to adenine (green), thymine (red), cytosine (blue), guanine (black). Pink and yellow marks indicate spontaneous and AA-induced deletion mutations, respectively.

The distribution of AA-induced and spontaneous mutations in the coding region of *gpt* is shown in Figure 5. The deletion was dominant (54%) and spread throughout the coding region with a preference for some sites, i.e., three out of 27 AA-induced deletion mutations occurred at positions 114 and 211. Moreover, two out of 23 AA-induced base substitutions with C to T transition occurred at position 211; therefore, these positions were considered the mutation hotspots for AA. Positions 38, 418, and 431 were also considered as other mutation hotspots for AA.

### Expression of *CYP2E1* in the Lung-Organoid

**FIGURE 6:**
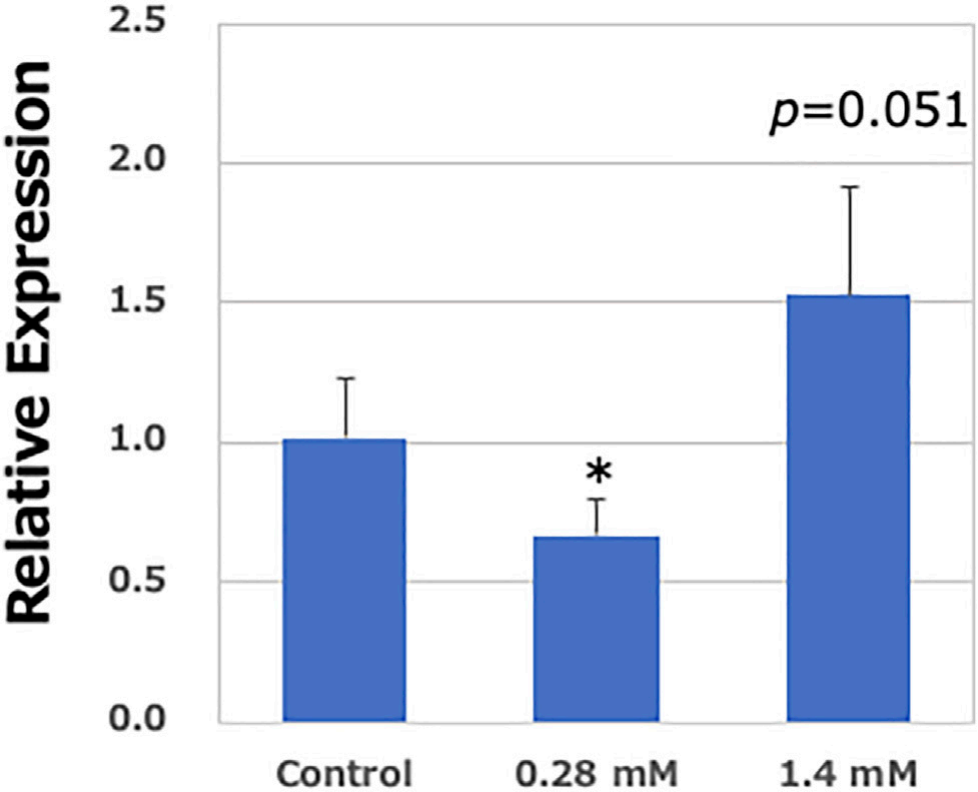
Relative expression levels of *Cyp2e1* in the lung-derived organoids. Real-time PCR analysis of mRNA expression of *Cyp2e1*. Values are set at 1.0 in untreated controls, and relative levels are expressed as mean ± SE (*n* = 6). GAPDH mRNA levels are used to normalize data. Significantly different from AA (−)/S9 (+) group at *p < 0.05.

According to a previous report, AA failed show a mutagenicity in an Ames test, regardless of the presence or absence of metabolic activation systems, due to insufficient *CYP2E1* expression in S9 fractions (International Agency for Research on Cancer (IARC), 1994). However, in the present study, a high concentration of AA clearly demonstrated mutagenicity. To confirm the expression of *Cyp2e1* mRNA, quantitative RT-PCR was performed. The expression of *Cyp2e1* was slightly increased in the pulmonary organoids than intact expression level in the lung tissues obtained from *gpt* delta mice (approximately 1/70th). A limited expression was observed in the pulmonary organoids. However, the mRNA expression level of *Cyp2e1* was significantly increased in the pulmonary organoids after exposure to a high-concentration (1.4 mM) of AA than that of vehicle control (only S9 mix exposure) organoids. In contrast, no obvious increase in *Cyp2e1* expression was observed at low concentrations (0.28 mM) of AA (Figure 6). Moreover, the expression of *Cyp2e1* was also induced by AA treatment in a concentration-dependent manner, without the S9 mix (Supplementary Figure S2).

## Discussion

Safety evaluation is generally required in the development of chemical substances; therefore, it is desirable to develop *in vitro* systems that can predict carcinogenicity in the short or medium term, considering the 3Rs (replacement, reduction, refinement). The *in vitro* genotoxicity tests have been widely used as simplified safety evaluation tests for chemical substances, but it is difficult to predict carcinogenicity from these tests alone. We recently established an *ex vivo* model for chemical carcinogenesis using normal mouse tissue-derived organoids and demonstrated its utility for detecting early molecular events in chemical carcinogenesis (Naruse et al., 2020). In the present study, we established an *in vivo* simulation assay platform for evaluating genotoxicity using organoids derived from *gpt* delta transgenic mice. We demonstrated that PhIP and AA showed strong genotoxicity in a dose-dependent manner in their target organs for carcinogenesis (Tables 1, 4).

In a similar experiment using liver-derived organoids as non-target tissues for carcinogenesis, PhIP showed an increasing tendency for genotoxicity in a concentration-dependent manner (Table 3). The background MF in the hepatic organoids was much higher than that in colonic organoids. However, a previous study showed that background MFs in the liver and colon are similar (Masumura et al., 1999). This disparity is possibly caused by different organoid preparations. Despite the high background MF in the hepatic organoid, it was almost comparable to that in colonic organoids. Moreover, the extent of formation of promutagenic DNA adducts, such as dG-C8-PhIP, was almost the same in the hepatic and colonic organoids after exposure to PhIP in the presence of S9 mix. In contrast, the MF in the liver in *gpt* delta mice treated with PhIP was significantly increased, but the values were approximately one-fourth of those observed in the colon (Masumura et al., 1999). Additionally, when PhIP was singly administered to F344 rats, the PhIP-DNA adduct level in the non-target tissue, the liver, was much lower than that in the target tissue, the colon (large intestine) (Schut and Herzog, 1992). In the *in vivo* study, the organ distribution after oral administration tended to have a significant effect on adduct formation, mutagenicity, and carcinogenicity. However, in the *in vitro* analyses in the present study, organ specificity, as observed in the *in vivo* study, was not observed because the organoids and PhIP were incubated together in the presence of metabolic activation systems. Therefore, the same degree of adduct formation and MFs were observed in both organoids derived from the target and non-target tissues for carcinogenesis.

The mutation spectrum analysis revealed that G:C to T:A and G:C to C:G transversions, G:C to A:T transition and deletion were dominant in the PhIP-exposed colonic organoids. Of these, G:C to T:A, G:C to C:G, and deletion were also dominant mutation spectra in the colon of *gpt* delta mice treated with PhIP (Masumura et al., 2000). Previous reports have indicated that dG-C8-PhIP leads to G to T transversion using a translational synthesis technique (Shibutani et al., 1999). However, the G:C > C:G transversion is a rare mutation induced by mutagens and carcinogens. It has also been reported that DNA adducts, such as spiroiminohydantoin and guanidine hydantoin produced by further oxidation of 8-OH-dG, are involved in the induction of G:C > C:G transversion (Kino and Sugiyama, 2001; Kornyushyna et al., 2002). Additionally, DNA strand breaks and 8-oxo-dG formation are induced during dG-C8-arylamine adduct formation *via* rearrangement of dG-N7-arylamine hydrazine adducts (Bellamri et al., 2021). Based on these chemical mechanisms, G:C to C:G transversion and deletion may be increased by PhIP exposure *in vivo* and *in vitro*.

The PhIP-induced mutations were spread throughout the coding region of *gpt*, with a preference for some sites. However, only some of the mutational hotspots observed in the organoids corresponded to the PhIP hotspots previously reported in an *in vivo* study (Masumura et al., 2000). This may be due to the effects of the dose and duration of PhIP exposure. In the study by Masumura et al., the animals were fed a diet containing 400 ppm of PhIP for 13-weeks; whereas, in the present study, the organoids were exposed only to 5 or 10 μM of PhIP three times, suggesting that mutations may be introduced in lesions where PhIP tends to form adducts and are difficult to repair. However, the proportion of these areas may increase with long-term exposure to high concentrations. Therefore, with an increase in the exposure of organoids to PhIP, the outcome would be similar to that of the *in vivo* study.

In contrast, exposure to AA also increased MF in the murine lung-derived organoids in a dose-dependent manner. Mutation spectrum analysis revealed that the proportion of G:C to A:T transition, A:T to T:A and A:T to C:G transversions, deletions, and insertions was statistically significant in the AA-exposed lung-derived organoids than *in vivo* historical data of control mice (Ishii et al., 2015). In contrast to the effect of PhIP, deletion mutation was the most prominent (54%), followed by G:C→A:T (16%). This trend is consistent with *in vivo* studies, i.e., single base pair deletion in 35.4%, followed by G:C to A:T in 16.7% (Ishii et al., 2015). Moreover, it has been reported that the highly reactive electrophilic epoxide, glycidamide (GA), is produced by the oxidation of AA by cytochrome P450 2E1 (CYP2E1), which is potentially involved in causing mutations in the genome instead of or concomitant with AA (Segerbäck et al., 1995; Sumner et al., 1999; Ghanayem et al., 2005; Tungeln et al., 2012). Several studies have demonstrated that A:T to G:C transition, and A:T to T:A, G:C to C:G and G:C to T:A transversions are induced by exposure to GA (Besaratinia and Pfeifer, 2004; Ishii et al., 2015; Manjanatha et al., 2015). Additionally, Zhivagui et al. analyzed the mutational signature of GA using primary mouse embryonic fibroblasts (MEFs) isolated from human-p53 knock-in (Hupki) mouse embryos. They found that A:T to T:A transversion was predominant and showed strong transcribed-strand bias in the 5′-CTG-3′ (complementary to 5′-CAG-3′) context (Zhivagui et al., 2019), suggesting that GA-DNA adduct formation, such as N3-(2-carbamoyl-2-hydroxyethyl)-adenine, may be involved in inducing mutations in this lesion. In the present study, in addition to the mutation spectrum associated with AA, a mutation spectrum derived from GA, such as A:T to T:A, was clearly observed, suggesting that GA was produced via the metabolism of AA in this experimental system. Moreover, it has been reported that the Ames test yielded negative results with AA regardless of the presence or absence of metabolic activation systems (International Agency for Research on Cancer (IARC), 1994). However, in the present study, a clear increase in MFs was observed upon exposure to a high concentration of AA. It has been suggested that a sufficient amount of *CYP2E1* is not present in S9 (metabolic enzymes) widely used in genotoxicity tests, such as the Ames test; therefore, AA may not convert to GA, which is the genotoxic derivative of AA, in *in vitro* systems. Despite using the same S9, a significant induction of the MFs was observed in the present study, suggesting that the mouse-derived lung organoids may have metabolic potency to produce GA from AA. To clarify this contradiction, we studied *Cyp2e1* expression in the lung organoids. The mRNA expression of *Cyp2e1* was significantly induced in the samples exposed to high-dose AA but not in those exposed to low-dose AA. Moreover, an increase in the MFs was not observed in low-AA exposure, whereas MFs were increased in high-AA exposure. Based on this observation, it is suggested that GA may be produced via metabolization of AA by CYP2E1, thereby inducing mutations in *gpt*.

In conclusion, we have established an *in vivo* simulation assay platform for evaluating genotoxicity using organoids derived from *gpt* delta transgenic mice. We have demonstrated that PhIP and AA show strong genotoxicity in a dose-dependent manner in their target organs for carcinogenesis. Additionally, the dominant mutation spectrum observed in chemically exposed organoids, i.e., G to T/A for PhIP and A to C/T for AA, corresponded to those observed in previous *in vivo* studies. Therefore, genotoxicity assay using organoids is an alternative technique for *in vivo* simulation of genotoxicity assay platforms. Further studies using a variety of other chemical substances are required to verify the applicability of the assay.

## Data Availability

The original contributions presented in the study are included in the article/Supplementary Material, further inquiries can be directed to the corresponding author.
