# Supplementary material for: Establishment of Novel Genotoxicity Assay System Using Murine Normal Epithelial Tissue-Derived Organoids
Source: Front Genet. 2021 Nov 18;12:768781. doi: 10.3389/fgene.2021.768781 (PMC8638810; doi:10.3389/fgene.2021.768781)

Supplementary Material

1 Supplementary Figures and Tables

1.1 Supplementary Figures

Supplementary Figure 1. Distribution of spontaneous and PhIP-induced mutations in the coding region of the *gpt* gene in hepatic organoids.

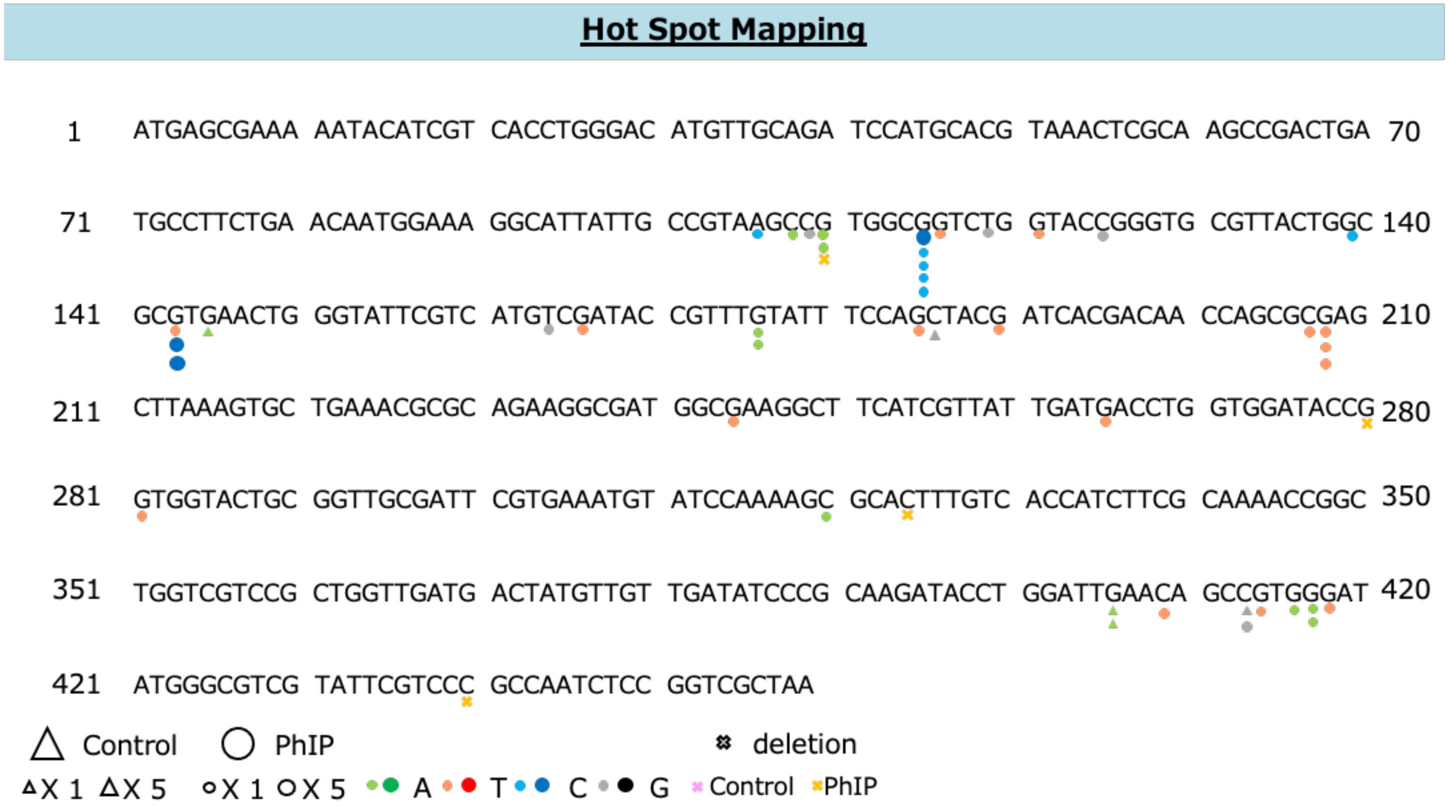

Supplementary Figure 2. Relative expression levels of *CYP2E1* in the lung-derived organoids in the absence of the S9 mix. Real-time PCR analysis is used to determine the mRNA expression levels of *CYP2E1*. In untreated controls, values are set at 1.0, and relative levels are expressed as mean ± SE ($n = 6$). *GAPDH* mRNA levels are used to normalize data. Data significantly different from the AA(-)/S9(+) group is indicated as *$p < 0.05$, **$p < 0.01$.

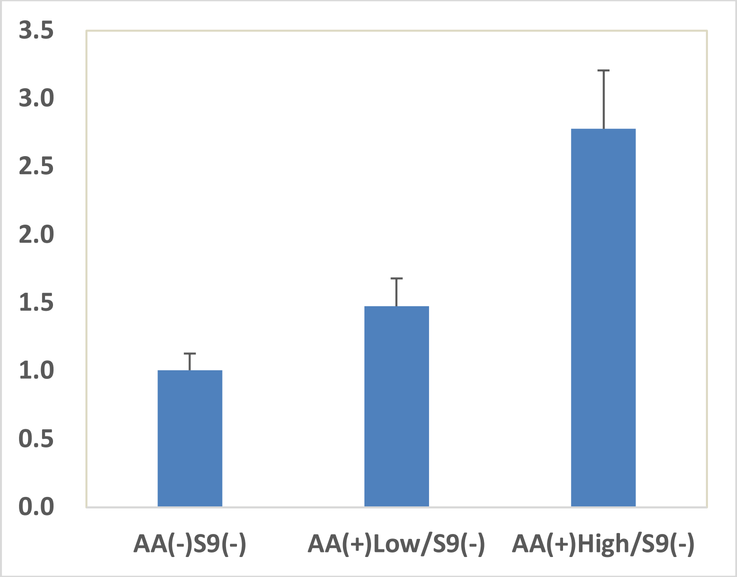

Supplement: Supplementary file 1 [file Presentation1.pdf]
